# Investigation of a Micro-test for Circulatory Autonomic Nervous System Responses

**DOI:** 10.3389/fphys.2017.00448

**Published:** 2017-07-21

**Authors:** Maximilian Moser, Matthias Frühwirth, Dietmar Messerschmidt, Nandu Goswami, Leopold Dorfer, Frank Bahr, Gerhard Opitz

**Affiliations:** ^1^Human Research Institute of Health Technology and Prevention Research Weiz, Austria; ^2^Institute of Physiology, Medical University Graz Graz, Austria; ^3^Austrian Society for Controlled Acupuncture Graz, Austria; ^4^German Academy for Acupuncture Munich, Germany

**Keywords:** Nogier reaction, heart rate variability, heart rhythm flexibility, acupuncture, autonomic nervous system, aging, diabetes, inflammation

## Abstract

**Aims and Objectives:** The autonomic nervous system plays an important role in homeostasis and organismic recreation, control of immune function, inflammation, and bone growth. It also regulates blood pressure and orthostasis via vagal and sympathetic pathways. Besides recording of heart rate variability (HRV), which characterizes medium (1–5 min) and long term (circadian) autonomic tone or modulation, no gentle tests of short-term autonomic reactivity and control are available. In 1976 Nogier described a short time cardiovascular response (“Réflexe Auriculo Cardiaque”, RAC) which could be used to investigate short term autonomic reactions without changing system characteristics and thus being repeatable in short intervals. In this paper, we investigated the possible application of the Nogier reaction as a micro-test for the identification of a disturbed sensitivity or reactivity of the autonomic nervous system.

**Methods:** We statistically analyzed cardiovascular signals derived during the application of small repeated stimuli utilizing methods of signal averaging to characterize the physiological background. Specifically, the Nogier reaction was investigated using simultaneous recordings of ECG, pulse waves, and respiration.

**Results:** Significant fast (delay 1–5 s) and slower (delay 6–12 s) cardio-autonomic responses to different stimuli which characterize short term were observed. From time characteristics and type of signals where they occur we deduce that fast changes observed in heart rate are vagal reactions to the small stimuli whereas slower changes observed in pulse waves stem from sympathetic nervous system responses.

**Conclusions:** The investigated autonomic micro-test opens the possibility to differentially investigate both limbs of the autonomic nervous system with minimal stimuli. It can be performed within seconds and does not change the set point of the system in opposition to less subtle tests such as Valsalva maneuver. Therefore, it is well-suited for quick, repeated measurements of autonomic nervous system reactivity.

## Introduction

Most cells in the body react to signals of the autonomic nervous system. This explains the importance of this unconscious part of our nervous system for homeostatic regulation and hemodynamic balance. A quick clinical test of the autonomic nervous system response would therefore be useful. Over the last few years, heart rate variability (HRV) measurements have been developed for this purpose (Moser et al., 1994, 1998; Task Force of the ESC and NASPE, 1996; Kralemann et al., 2013). Cardiac autonomic tone or modulation (Parati and Di Rienzo, 2006) as well as its changes due to physical or psychological load has been assessed using this non-invasive method. However, the time needed to obtain reliable values for HRV has been found between 5 min and 24 h, which is rather long if questions related to the patient autonomic reactions need to be answered fast. For this reason, shorter, repeatable tests, which allow a rapid micro-testing of the ANS response to different stimuli should be investigated. Searching for a suitable test for the organismic response to acupuncture, Nogier discovered a cardiovascular reaction, which he could trigger by slightly stimulating certain points mechanically on the human ear (Nogier, 1972). He called it “*réflexe auriculo cardiaque*” or RAC (Oleson, 2002) without being able to give sufficient physiological explanation or an autonomic regulatory background. Later Bahr developed this method and used it as a diagnostic test in acupuncture (Bahr and Strittmatter, 2010). Indeed, the Nogier reaction has found its way to the Chinese mainland acupuncture textbooks today (Bahr and Strittmatter, 2014). Medical doctors utilizing the Nogier reaction report that even minor disturbances of the autonomic nervous system can be observed.

In this paper, for the first time, we statistically analyze cardiovascular signals derived during the repeated application of small physiological stimuli as used in Nogier reaction testings, utilizing methods of signal processing, and averaging to highlight a possible physiological explanation of the reaction and make it measurable by scientific methods. We further investigated the possible application of the Nogier reaction testing for the identification of functional disorders evoked by a disturbed sensitivity or reactivity of the autonomic nervous system. We believe that the results of our study are important for bedside identification of functional disorders.

## Methods

### Participants and stimuli

Healthy participants (*n* = 20; 11 males), aged 19–47 years were subjected to five different stimuli during a period of 20 min (Table 1). Stimuli had been selected to be either below or at the border of conscious sensory perception, to produce as little disturbance of the ANS as possible, except for the clapping. To prevent anticipation of the following stimulus, the stimuli were applied in a fixed but irregular sequence. For statistical analyses purposes, each treatment was repeated 20 times. During experimentation, the participants rested in a supine state with eyes closed.

**Table 1:** Participant characteristics—mean ± SD or absolute frequency.

	**Participant data**
Age (years)	27.3 ± 7.8
Height (cm)	175.7 ± 10.0
Weight (kg)	68.3 ± 10.2
BMI	22.1 ± 2.2
Sex	11 male, 9 female
Systolic pressure (mmHg)	131.1 ± 12.5
Diastolic pressure (mmHg)	82.6 ± 9.8
Handedness	16 right-handed 3 left-handed 1 both

#### Stimuli applications

The stimuli applied to the participants are described in Table 2.

**Table 2:** Applied stimuli in the order of application.

**Short name**		**Explanation**
Infrared laser	IL	Infrared laser (wavelength 830 nm, 30 mW, MODULAS, Pierenkemper) directed to the center of the forehead without touching the skin, was silently switched on for 2 s
Electric field	EF	A direct current electric field (3 V) generated by pin electrodes pointing in opposite directions, tip distance 5 cm (“3V-hammer,” Pierenkemper, Wetzlar) brought 1 cm above the forehead between the brows
Sham laser	SL	The same laser as above was directed to a neutral skin spot at the forehead without touching the skin and without being switched on
White light	WL	Low level white bulb light (mini 3000, Heine, Herrsching) directed at the cheek without touching the skin was silently switched on for 2 s
Clapping	CL	Hand clapping above the patient's head, eyes closed (this was the only treatment that led to a strong conscious perception of the stimulus)

*Typical interventions used for testing the Nogier reaction by acupuncturists were chosen like EF, WL and IL. Additionally a sham stimulus (SL) with no anticipated effect and a strong acoustic stimulus (CL) were chosen to verify the reactivity of the Nogier reaction*.

The control intervention for all others was the sham laser (SL), which was presented in the middle of the experiment. During SL, the laser head was directed to a neutral non-acupuncture spot a few centimeters away from the IL spot and not switched on.

Due to the homeostatic properties of the ANS, we expected an autonomic response of either the sympathetic or the parasympathetic branch or of both to occur after the applied stimuli. The strength of the stimuli thereby should be reflected in either vagal withdrawal (low stimuli like infrared Laser IL, Light stimulus WL, and electric field EF) or sympathetic activation (strong stimuli: clapping CL). No reaction was expected following Sham Laser SL.

### Experimental procedure

A polysensoric sensor jacket originally developed for a joint Austrian–Russian Space mission (AustroMir) was used to record the data (Moser et al., 1992; Gallasch et al., 1996, 1997). The equipment was technically updated for this study. Before the experiments, participants were made familiar with the sensor jacket used for measurements. The physician informed them that small non-painful stimuli without skin contact would be applied during the experiments for a total duration of about 20 min. Participants relaxed in a supine position for 15 min before commencement of the treatments.

During the experiment the participants were asked to keep their eyes closed and to relax. The applications of the stimuli were timed by an additional palmtop (Hewlett Packard, HP150LX), which was programmed to indicate the time of stimulus applications with varying sequences of intervals between 12.5 and 17.5 s, visualized by a progress bar. It was visible only to the physician. After the experiment, the participants were asked whether they had perceived the stimulus applications consciously.

### Ethical standards

Experiments complied with the laws of Austria, the country where the experiments were performed. Participants gave their written consent to participate in the study. The study was conducted according to the guidelines of “good clinical practice” (ICH-GCP), the Helsinki Declaration as well as data protection stipulations. The measurement procedures and methods applied were non-invasive and approved according to CE guidelines.

### Physiological variables and measurement equipment

**Figure 1:**
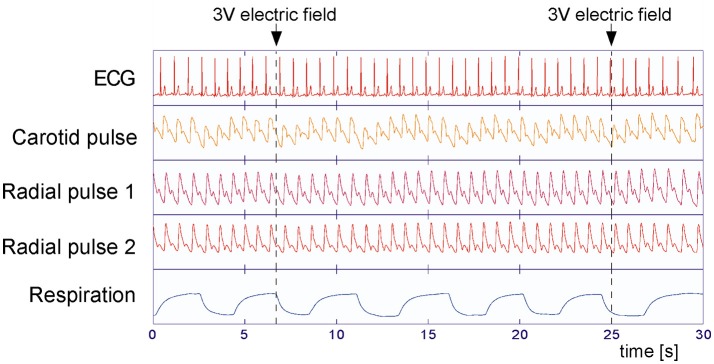
Measured variables during the experiments. Electrocardiogram (ECG), carotid, and radial arterial pulses (P2 at CUN and P1 at GUAN position of traditional Chinese medicine, Tang et al., 2012) and respiratory air flow were recorded simultaneously at 200 Hz (ECG: 1,000 Hz) sampling rate. Little influence of the applied stimuli (arrows) on the measured variables can be observed, but a clear respiratory effect is seen.

Ten minutes preceding and during the application of the stimuli, polysensory measurements using the sensor jacket connected to a portable data collection device were performed (Human Research Institute, www.humanresearch.at; Moser et al., 1992; Gallasch et al., 1996, 1997). Simultaneously seven primary signals were recorded: electrocardiogram (ECG), pressure pulses at carotid and optical pulses at two locations at the right radial artery (see below), respiratory air flow (Figure 1), and accelerometry signals at two locations. The pulse locations selected were on (P1) and one centimeter distal (P2) of the apophysis radii (P1 corresponding to the Cun resp. P2 to the Guan position in Chinese pulse wave diagnosis), All parameters were recorded at 200 Hz, 16 Bit, except ECG, which was recorded at 1,000 Hz, 16 Bit. Instantaneous high precision inter-beat-intervals were computed at a time resolution of 1 ms.

**Figure 2:**
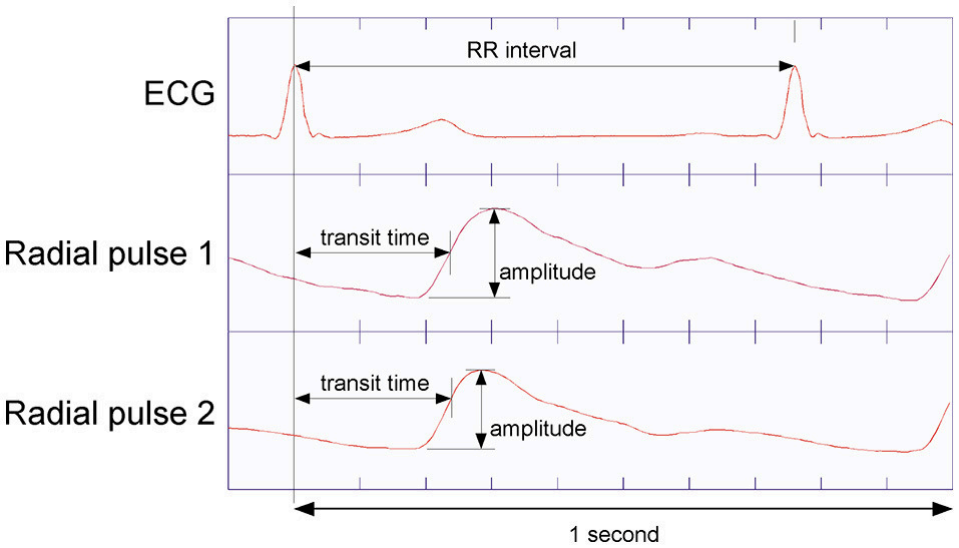
Determination of pulse transit time as the time interval from ECG R-peak to steepest ascent of the pulses recorded at the P2 resp. P1 position at the radial artery (Tang et al., 2012). Pulse Amplitude is from bottom to the peak of respective pulse waves.

Parameters selected for further calculation were: RR-interval, pulse transit time (the time between R-peak and the steepest ascent of pulse wave; Moser et al., 1992) at two locations at the wrist radial artery, and the amplitude of the pulse waves on these locations (Figure 2). From the primary data of pressure pulses, pulse wave transit time to carotid and two right radial artery locations were computed, using the time from R peak to steepest ascent of respective pulses (Moser et al., 1992).

Due to frequent artifacts (e.g., swallowing or movements) the carotid pulses and the accelerometer signals were not analyzed further.

### Data preprocessing

The original signals were printed and visually inspected. Isolated non-physiological values were marked as artifacts and replaced with the average of the values before and after the artifact. If more than one artifact occurred between two stimuli or if the whole series was locally corrupted those signals were excluded from further processing. Overall, 5.6% of the data were excluded for this reason.

**Figure 3:**
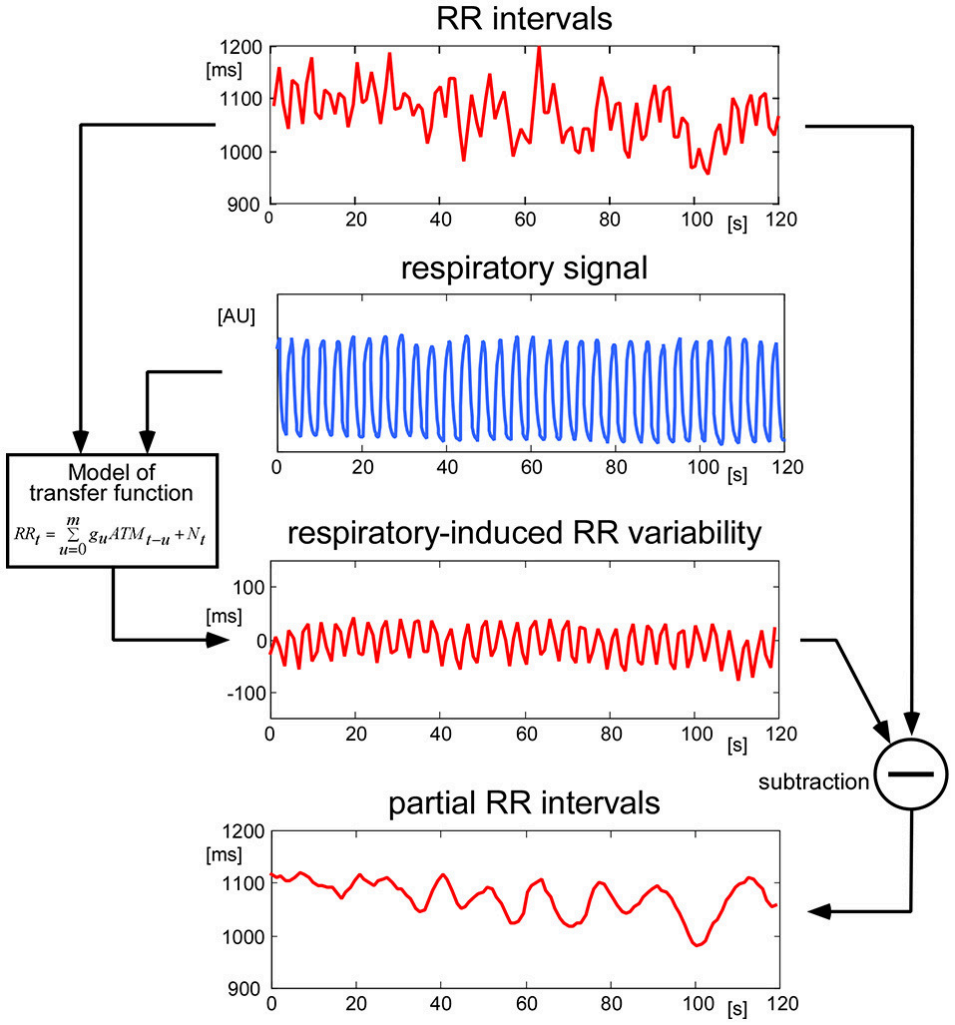
Handling of respiratory modulation of the RR intervals. To remove disturbing oscillations originating from respiratory modulation, a continuous transfer function between the respiratory signal and the RR intervals was computed. The estimated modulation of RR due to respiration was then removed from the original RR signal. The same procedure was performed for all physiological signals before performing further analyses.

To minimize the effects of respiratory modulation on the physiological variables, a transfer function between the recorded respiratory air flow and each of the other parameters was computed by a method modified from Florian and Pfurtscheller (1997). This transfer function was determined piecewise throughout the measurements. The fraction of the target signal induced by respiratory modulation was estimated using this transfer function and subtracted from the original signal (Figure 3). The residual signal was fed into further analysis.

## Statistical analysis

To characterize and analyse the stimulus responses, 20 consecutive heartbeats were used: 7 pulses before the stimulus, the pulse next to the stimulus, and 12 pulses after the stimulus. For each of these series of 20 consecutive beats the average of the seven baseline values was subtracted from the time series to get rid of data offsets.

For statistical analysis, the mean of the (at most) 20 stimulus responses for each participant, each of five interventions and five measurement parameters (RR-intervals, two pulse wave amplitudes, two pulse wave transit times) was used. From the resulting 500 time series (1 × 20 × 5 × 5), 20 were missing: 10 P1 pulse recordings (five run-time, five amplitude series) from three participants, and all P2 pulse recordings from one participant (10 series). These 20 (or 4%) were replaced by the constant mean value of all other recordings, adopting a statistically conservative strategy.

A multivariate analysis of variances (MANOVA) for repeated measurements was applied for each stimulus separately. The time series, the seven pulses before and 12 pulses after the stimulus, were treated as a within participant factor. As this results in five overall tests, a level for *p* < 0.01 was chosen to be significant to achieve a conservative overall acceptance level of 0.05. For each physiological parameter which showed a significant change over time *post hoc* tests (Student's *t*-test) were performed. All calculations were performed using SPSS.

## Results

All participants displayed physiological signals without arrhythmias and with few artifacts. Figure 1 shows a representative 30 s polygraphic tracing of original data like ECG, pulses and respiration before, during and after two stimulations with the same stimulus in a 26-year-old male participant. The stimulus, a 3 V electric field (EF) brought close to the participant's forehead, does not produce a visible effect in the raw data recording. A clear baseline and amplitude modulating effect of respiration is visible in ECG and pulse recordings; a reason why the timing of stimuli in an irregular way is important (to avoid disturbing influences of respiration). Subtracting the respiratory modulation as described in the methods resulted in a marked reduction of periodic respiratory oscillations in inter-beat intervals, pulse transit times, and pulse amplitudes, as seen in the example of Figure 3 (above).

After subtracting the respiratory effects, we aligned the signal snippets seven pulses before to 12 pulses after the trigger stimuli, and averaged them for each stimulus separately. The procedure is similar to averaging signals of cortically evoked potentials following arousal stimuli (Borsanyi and Blanchard, 1962). All stimuli except hand clapping (CL) were not perceived consciously by the participants, according to their own declaration.

**Figure 4:**
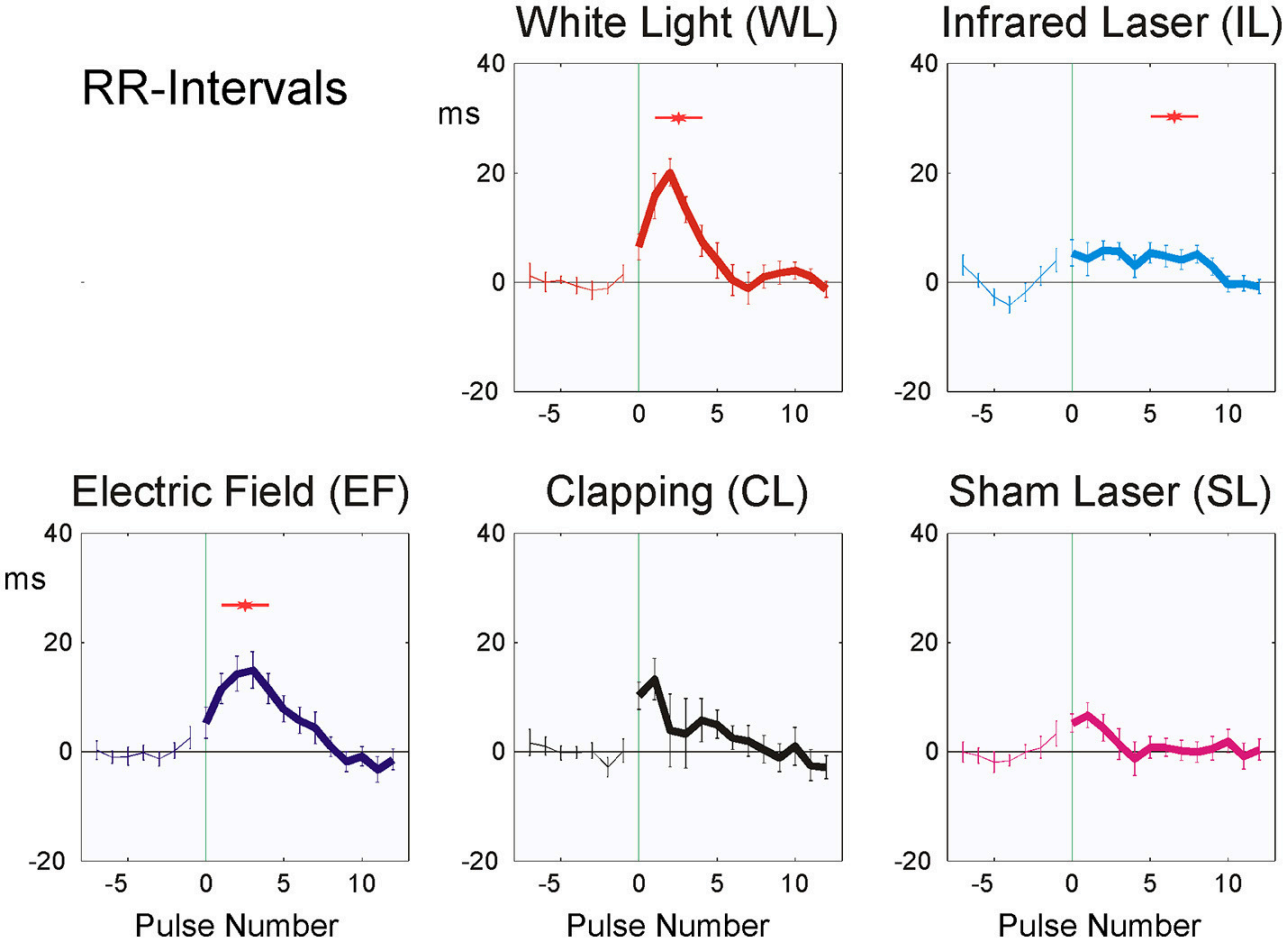
Averaged stimulus responses of RR-intervals (20 participants). The difference from the mean of the last seven pulse intervals prior to stimulus application is plotted. Stimuli were applied at time zero, indicated by vertical green thin line. Abscissa: No. of beat before/after stimulus, ordinate: RR interval (ms, mean ± SEM). A total of 392 sequences were used for each figure. Red asterisks show post hoc t-tests of the total sample against zero with *p* < 0.01.

Averaging over all experiments of the 20 participants, all of the applied stimuli, with the exception of SL, resulted in an initial prolongation of the heartbeat intervals 1–5 after the stimulus (Figure 4) by 5 (IL) to 20 (WL) ms. This indicates a fast vagotonic response to the stimuli, as vagal control of the heart is faster than sympathetic one, due to properties of the vagal synapses (Warner and Cox, 1962).

The amplitude of the physiological reactions was largest in the white light exposure (WL), small or no reaction could be seen in the laser stimulus experiments (SL, IL).

**Figure 5:**
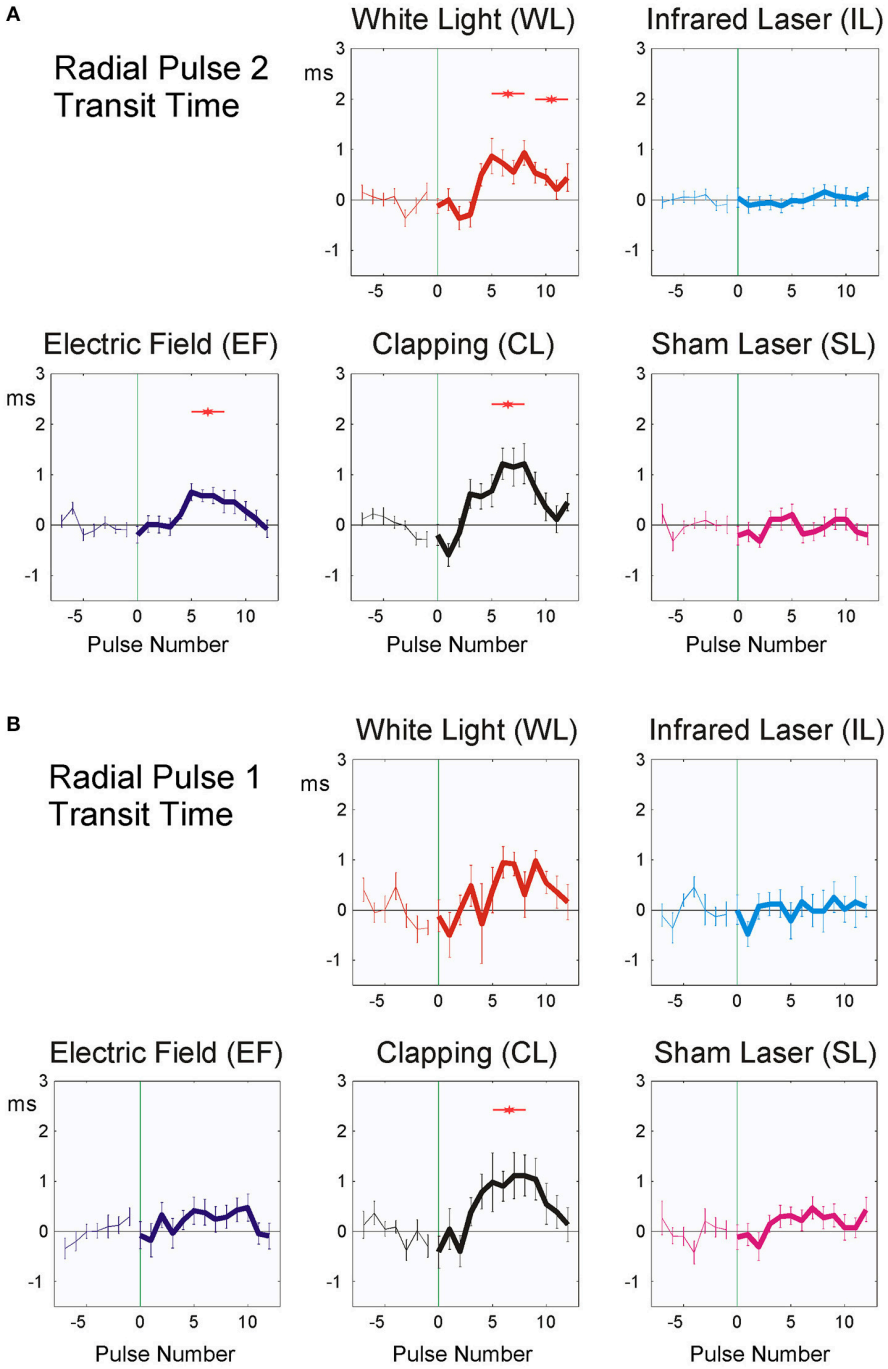
Averaged stimulus response of P2 (19 participants, **A**) and P1 pulse transit time (20 participants, **B**). The difference from the mean of the last seven pulse intervals prior to stimulus application is plotted. Stimuli were applied at time zero, indicated by vertical green thin line. Abscissa: No. of beat before/after stimulus, ordinate: Pulse transit time (ms, mean ± SEM). A total of 392 (P2) resp. 372 (P1) sequences were used for each figure. Red asterisks show post hoc t-tests of the total sample against zero with p < 0.01.

P2 pulse transit time showed significant responses in EF, WL, and CL (Figure 5A and **Table 4**), P1 pulse transit time (Figure 5B) remained more or less stable after stimulation, except after CL.

**Figure 6:**
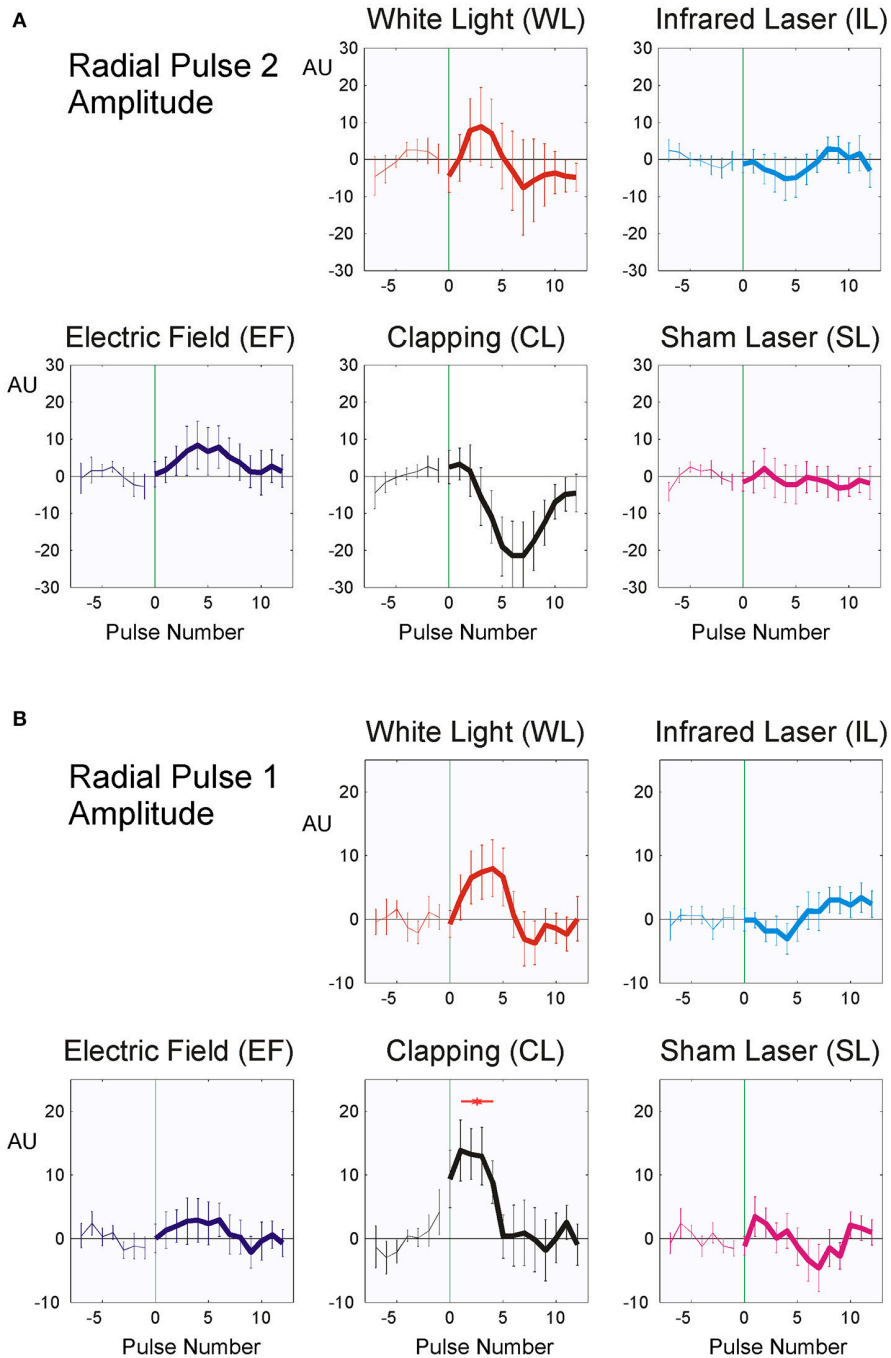
Averaged stimulus response of P2, (19 participants, **A**) and P1 pulse amplitude (20 participants, **B**). The difference from the mean of the last seven pulse intervals prior to stimulus application is plotted. Stimuli were applied at time zero, indicated by vertical green thin line. Abscissa: No. of beat before/after stimulus, ordinate: pulse amplitude (Arbitrary units, mean ± SEM). A total of 392 (P2) resp. 372 (P1) sequences were used for each figure. Red asterisks show post hoc t-tests of the total sample against zero with *p* < 0.01.

As noted in the methods above, “clapping” causes significant reactions in all parameters except RR intervals (Table 3; *p* < 0.02; η^2^ = 0.119), whereas the sham stimulus (SL) showed no significant physiological reaction (*p* > 0.15; η^2^ = 0.050). A lower stimulus response to Laser (η^2^ = 0.058) was observed. The RR intervals reacted most sensitively to the stimuli and the reaction was significant with all three minimal interventions, but not with clapping and SL (η^2^ = 0.135). P2 and P1 pulse amplitudes (Figures 6A,B) in contrast showed significant reactions only to the strongest stimulus of hand clapping CL.

**Table 3:** Results of the (M)ANOVA analysis of the physiological reactions to the applied stimuli.

***N* = 20**	**Parameter (univariate p-values)**					**Multivariate**	
**Stimulus**	**RR intervals**	**P2 transit time**	**P2 amplitude**	**P1 transit time**	**P1 amplitude**	**Overall (*p*)**	**η^2^**
Laser (IL)	**0.000**	0.995	0.927	0.903	0.482	0.219	0.058
Electric field (EF)	**0.000**	**0.000**	0.836	0.293	0.920	**0.000**	0.107
Sham laser (SL)	0.152	0.483	0.997	0.367	0.481	0.644	0.050
Light stimulus (WL)	**0.000**	**0.000**	0.907	0.047	0.047	**0.000**	0.125
Clapping (CL)	0.012	**0.000**	**0.000**	**0.000**	**0.001**	**0.000**	0.119
Multivariate (*p*-value)	**0.000**	**0.000**	0.323	**0.000**	**0.000**	**0.000**	0.050
η^2^	0.135	0.077	0.010	0.031	0.024		

Deviations from the state before stimuli are in bold print, if p-values are lower than 0.01. Neither group-effects (between factor), nor interactions time ^*^ group were observed (all p > 0.05; not illustrated). An effect size is given as partial eta-squared value (η^2^) for each stimulus and parameter, which is analogous to r^2^ (the percentage of explained variance for the stimulus response).

## Discussion

We report here for the first time a systematic scientific investigation of the Nogier reaction or RAC, a cardio-autonomic micro-reaction described first by Nogier (1972). This reaction is already utilized as an indicator of autonomic reactivity of patients in acupuncture (Bahr and Strittmatter, 2010). As a non-invasive and repeatable test of ANS function, it has the potential to become a common diagnostic micro-test of autonomic nervous system performance. As the ANS is adaptive, we tried to choose small stimuli that do not change the set-points of the ANS for longer than a few seconds. The stimuli should be large enough to get a small transient reaction of the ANS, but small enough to not disturb the set-points.

Significant and reproducible cardio-autonomic reactions to different stimuli were observed in this study. These can be useful in the characterization of short-term autonomic responses to small stimuli. Central (seen in RR interval changes except in CL and SL stimuli) as well as peripheral cardiovascular responses (visible in changes of pulse transit time and/or amplitude in CL, EF, and WL) indicate that the observed effects result in immediate vagal as well as delayed sympathetic reactions involving the heart and the peripheral circulation. This opens the possibility to differentially investigate both limbs of the autonomic nervous system with very small stimuli, which are mostly below the threshold of consciousness. To obtain artifact-reduced recordings of the changes due to the stimuli applied, a model had to be developed to subtract the influences of respiration (Figure 3). Only after this reliable stimulus responses are obtained from the data.

Short-term heart rate changes proved to be the most common statistically significant reaction (Figure 4 and Table 3). A high resolution and undisturbed heart rate recording (in our case 1,000 Hz; 1 ms, 16 Bit) is necessary due to the small effects observed (5–20 ms; see Figure 4). In the autonomic nervous system, vagal response to a stimulus is faster and more pronounced if the stimulus is small and short (Schandry, 1989). Only larger and longer stimuli are able to activate strong sympathetic responses. There are two possible reasons for this:

– Sympathetic activation is more demanding for the body than vagal withdrawal, although both types of reaction result in similar heart beat acceleration. Strong sympathetic activation also increases the risk for myocardial fibrillation (Moser et al., 1994).
– Physiological investigations have shown that the sympathetic synaptic processes are slower than the parasympathetic ones: sudden activation of sympathetic cardiac fibers show a significantly slower rise and slower fall than their vagal counterparts (Kirchheim, 1982; Lehofer et al., 1997, 1999; Moser et al., 1998).

In this study, we found mainly fast (in the RR intervals) as well as some delayed reactions (in the pulse wave amplitudes and transit-times) following different stimuli. We assume that the earlier correspond to vagal influences whereas the latter to sympathetic reactions of the autonomic nervous system.

The observed fast prolongation of the inter-beat intervals in WL and EF corresponds to an initial significant decrease in heart rate due to the reaction and to a transient increase in vagal tone (as shown in Figure 4 and Table 4). This is the first phase of the Nogier reaction, which then is followed by a second one observable in pulse transit times and pulse wave amplitudes (Figures 5, 6 and Table 4). Vessel wall tension and peripheral resistance are both responsible for such later changes (Kenner, 1996). Both are of sympathetic origin and show a longer reaction time than the corresponding heart rate changes deriving from vagal sources, which can be seen insignificant mid-time rather than early responses (Table 4) in pulse wave amplitudes and pulse transit times. Fast vagal responses observable in heart beat intervals (beats 1–5, Figure 4) are barely or not visible in the pulse transit times (Figure 5), but can be seen in pulse amplitude (Figure 6), at least in white light stimulus (WL) and clapping (CL). Maybe this lack of vascular reaction can be explained by the mostly sympathetic but not vagal innervation of peripheral resistance vessels. On the other hand, the later reaction of the resistance vessels, visible in the increase in pulse transit time (beats 4–10; Figure 6, WL and CL) cannot be seen in the RR Intervals (beats 4–10, Figure 4), so the later (sympathetic) reaction is mainly restricted to vessels and does not involve heart-beat intervals. This is especially evident in CL, where only a significant peripheral reaction was observed. The ability of heart rate variability measures alone to separate parasympathetic and sympathetic modulations is limited (Billman, 2013), and the presented method using additional pulse signals might help to solve the problem of characterizing sympatho-vagal balance.

**Table 4:** *Post hoc* tests of early (pulses 1–4 after the stimulus), intermediate (pulses 5–8), or late (pulses 9–12) responses.

***t*-Test (*N* = 20)**		**RR intervals**	**P2 pulse transit time**	**P2 pulse amplitude**	**P1 pulse transit time**	**P1 pulse amplitude**
Infrared laser	Early response	0.011	0.449	0.510	0.815	0.319
	Intermediate resp.	**0.006**	0.695	0.715	0.949	0.619
	Late response	0.728	0.550	0.913	0.619	0.150
Electric field	Early response	**0.000**	0.638	0.276	0.697	0.444
	Intermediate resp.	0.039	**0.000**	0.280	0.125	0.571
	Late response	0.281	0.231	0.717	0.231	0.759
Sham laser	Early response	0.170	0.704	0.977	0.883	0.416
	Intermediate resp.	0.730	0.834	0.763	0.012	0.386
	Late response	0.728	0.843	0.455	0.119	0.733
White light	Early response	**0.000**	0.833	0.474	0.824	0.109
	Intermediate resp.	0.673	**0.001**	0.720	0.012	0.962
	Late response	0.447	**0.003**	0.430	0.007	0.598
Clapping	Early response	0.195	0.637	0.631	0.463	**0.007**
	Intermediate resp.	0.239	**0.005**	0.030	**0.009**	0.923
	Late response	0.541	0.049	0.121	0.123	0.999

*p-Values vs. baseline are shown (all results p < 0.01 are in bold print if the corresponding factor combination was significant also in the overall test, cf. Table 3)*.

Three of the stimuli applied [laser (IL), electric field (EF), white light (WL)] in this study were selected from typical procedures used by acupuncture practitioners during Nogier reaction testing (Bahr and Strittmatter, 2010). They were complemented by two stimuli, which were supposed to produce no effect (SL) or a strong effect (CL). To prevent carry-over effects, we did not use a random sequence of the stimuli, but rather an anticipated order (from suspected weaker to stronger responses).

**Figure 7:**
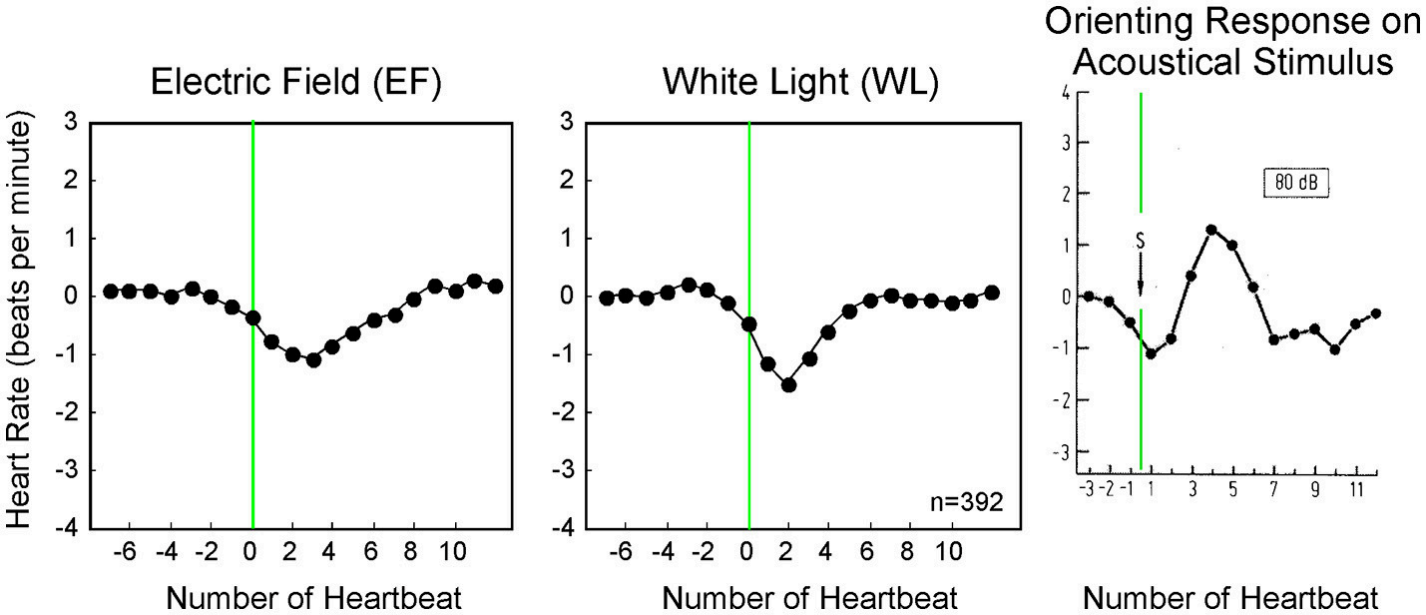
Averaged heart rate responses (392 stimuli per application) to two weak stimuli compared to a published orienting response from literature (d, Schandry, 1989). Whereas, the orienting response to a sudden 80 dB sound is biphasic, the smaller stimuli used in our experiment display a monophasic response.

In this study, the applied strength of all stimuli, except hand clapping, were selected below or at the level of conscious sensory perception. From the already well-investigated orienting reaction (OR) it is known that thalamic structures in the brain determine whether a stimulus is perceived consciously or not (Schandry, 1989). The OR shows similarities in the time course as well as the shape to the Nogier reaction described in this study (Figure 7). The OR is an autonomic response to stronger stimuli like audible 80–120 dB white noise or a visible light flash. The stronger stimulus of the OR results in a biphasic reaction; the Nogier reaction, however, usually is monophasic (Figures 4–7). Especially stronger stimuli (like 120 dB Sound) result in an initial acceleration of the heart (vagal withdrawal), whereas an initial deceleration was observed in our experiments (vagal release). It seems, therefore, likely, that the Nogier reaction is comparable to the orienting reaction for stimuli, but at the border of conscious perception. As such, the Nogier reaction might become a valuable tool to investigate small autonomic nervous system responses not observable with other methods. It might help to identify participants with subtle disturbances in autonomic regulation, like seen in early diabetes or other neuropathic conditions.

Not only in disease, but also in healthy subjects the ANS plays a role more important that previously assumed. Recent research has documented the importance of the autonomic nervous system for tasks like immune functioning or bone growth control, beside the long-known energy and humoral regulation. ANS control also is essential for orthostatic stability and baroreflex control (Moser et al., 1992) as well as neuromuscular control in microgravity (Gallasch et al., 1997). Autonomic receptors found at the cell surface of leucocytes have been traced during “information exchange” with autonomic nerve terminals along the wall of small blood vessels (Felten and Olschowka, 1987; Kin and Sanders, 2006). Concerning inflammation, a co-factor of many lifestyle diseases, an “inflammatory reflex” (Tracey, 2002) has been identified involving tissue located vagal afferents and efferents: macrophage cells in inflamed areas of the tissue produce inflammation signals, e.g., TNF alpha and interleukin-1 (Andersson and Tracey, 2012; Olofsson et al., 2012). These cytokines in turn attract and activate other lymphocytes from the nearby blood vessels. Vagal afferents carry receptors for these signals as well and “understand” the language of the immune cells reporting the inflammation location and strength to hypothalamic areas (Rosas-Ballina and Tracey, 2009). There this information is processed and vagal efferents leading to the inflamed area and to the spleen are activated (Olofsson et al., 2012). Nicotinergic acetylcholine receptors have been identified on the surface of macrophage cells. Upon vagal nerve stimulation and acetylcholine release, these receptors immediately down-regulate their cytokine production (Wang et al., 2003). This inflammatory reflex loop prevents over-activity of the immune system and allows the brain to locally control immune activity. It also represents the “first line” of inflammation control (Olofsson et al., 2012).

Disturbances of the vagal inflammation reflex are suspected to be responsible for a couple of diseases induced by chronic inflammation, including atherosclerosis, ulcerative colitis, Hashimoto disease, type 2 diabetes, and cancer (Medzhitov, 2010). Vagal modulation is reduced in most of these diseases (Huston and Tracey, 2011). Taken together, these findings suggest a common language between autonomic nervous and immune system and emphasize the role of the autonomic nervous system in immune responses.

With respect to the sympathetic system, adrenoceptors have been identified on the osteoblast cell surface. These receptors are able to block osteoblast activity and proliferation (Patel and Elefteriou, 2007). This may be one of the reasons why stress promotes osteoporosis. Another support to this hypothesis stems from the fact that beta blockers obviously prevent bone fractures (Reid, 2008). The sympathetic nervous system also influences pain perception, which is increased in nervous subjects (Liebmann et al., 1994, 1998; Lehofer et al., 1998).

In clinical practice patients present with subclinical disturbances of sleep quality and/or personal well-being. In such cases, although patients often show no manifest organic deviations, the patients' life quality may be markedly decreased. These states may indeed be precursors to later organic diseases. Functional disorders may even be associated with altered tone or reactivity of the sympathetic and parasympathetic nervous system (Thayer and Lane, 2007), the main components of the autonomic nervous regulation required for organismic function (Moser et al., 2006, 2008). On the other hand, patients with clear diagnoses might resist conventional therapy. Disturbances in the autonomic balance might be responsible for such disease resistance (Thayer and Lane, 2007).

The intention of this study was to investigate a micro-invasive tool for testing the ANS with very small stimuli based on a reaction described by Nogier and used in acupuncture diagnosis (Nogier, 1972). We developed a standardized measurement protocol that makes the testing tool easily usable and allows scientific recordings of the phenomenon. The detailed pathways from stimulus source to the ANS reaction vary between stimuli and may be complex. We did not yet focus on individual patient characteristics. To prove efficacy or diagnostic value of the entire procedure, further studies will be necessary. These studies should investigate the applicability of the Nogier test to different autonomic conditions like e.g., orthostatic instability, volatile hypotension, autonomic failure, and autonomic neuropathias. If it can be shown, that this micro-test allows an earlier diagnosis of these condition and possible improvements due to therapeutic interventions, the method could provide a valuable tool for early diagnosis and for therapy monitoring.

In summary, the recording of Nogier reaction, using subtle stimuli near the border of perception, might allow a repeated testing of the immediaty vagal as well as the delayed sympathetic reactivity.

## Clinical applications of the Nogier reaction

Vagal tone, expressed in modulation of heart beat, has been recognized recently as an important part of the inflammation control loop (Tracey, 2002). Vagal activity is reduced in chronic inflammatory conditions, where resolution of inflammation does not work, although the inducer of inflammation is no longer present. In some of these conditions, a positive feedback loop seems to exist, connecting inflammation and the pathology it accompanies. This vicious circle can be observed e.g., in some forms of obesity, which can lead to inflammation, whereas chronic inflammation can promote obesity and associated diabetes in return (Medzhitov, 2010). A non-functional vagal control loop might be a co-factor of diseases that are largely contributing to the morbidity and mortality in modern societies: cardiovascular failure, metabolic syndrome and cancer.

It is thus important to develop simple short- and long-term tests of the autonomic nervous tone and reactivity. While heart rate variability has been used to assess medium- or long-term testing (Axelrod et al., 1987; Moser et al., 1994; Task Force of the ESC and NASPE, 1996), there are few vagal test which could characterize the short term and low-level vagal sensitivity and reactivity of subjects (Moser et al., 1994; Flatt and Esco, 2016). We believe that the Nogier reaction could be used as an innovative and easy-to-do micro-test for autonomic nervous system sensitivity and reactivity. This is particularly important in the light of the new findings concerning the association of the vagus and inflammation.

## Ethics statement

This study was carried out in accordance with the recommendations of the WMA and with written informed consent from all subjects. All subjects gave written informed consent in accordance with the Declaration of Helsinki.

## Author contributions

MM: conceptualized the research, carried out measurements, interpreted, and wrote the manuscript; MF: processed the data, wrote software for calculations and graphics, helped with statistics; DM: carried out measurements; LD: data interpretation, manuscript writing, and recruited participants; NG and FB: data interpretation, manuscript writing; GO: conceptualized the research, planning of experiments, data interpretation.

### Conflict of interest statement

The authors declare that the research was conducted in the absence of any commercial or financial relationships that could be construed as a potential conflict of interest.
